# New Drugs, Appliances, and Things Medical

**Published:** 1892-08-06

**Authors:** 


### NEW DRUGS, APPLIANCES, AND THINGS MEDICAL.

### DISINFECTANTS.—CARBOLIC ACID AND ITS PREPARATIONS.

Calvert and Co., Manchester.

According to Squire's "Companion to the B.P.," carbolic acid is prepared in a crude state by treating certain tar oils heavier than water with a dilute solution of caustic soda, subsequent separation of the crude carbolic acid from its alkaline solution by the addition thereto of a mineral acid, usually sulphuric. The crude carbolic acid thus obtained is submitted to fractional distillation and crystallisation, with other purification processes, having for their object the entire removal of the last traces of cresylic and other tar acids and bases and sulphur compounds, &c. The chief tests, according to the same authority, are: 1. Its odour, which is characteristic. 2. The melting-point, which should not be below 91·5 deg. Fah. 3. Its solubility in water, 1 in 12—18. It coagulates albumen, does not redden litmus, nor does it affect a ray of polarised light. It combines with metallic bases. Its uses are all of an antiseptic nature. It acts by destroying germ life and as an anti-putrescent. Its drawbacks are that it is poisonous, and this in varying degrees. It is directly in concentrated solution an escharotic destructive poison. Messrs. Calvert, as we noticed some time back, have adopted a special form of cork for their bottles, so that no one can possibly open them without becoming aware of the difference between them and other bottles. A more chronic form of poisoning takes place in certain patients, where it is used for a long period as a dressing for extensive wounds. The urine becomes smoky or dark, gives off an odour of carbolic acid, and reveals its presence in other ways. Notwithstanding this, it is a fact that in some form or another carbolic acid is one of the most extensively used disinfectants and antiseptics. These varying forms we shall notice briefly, and have requested Messrs. Calvert, of Manchester, as the chief manufacturers of carbolic acid, to enable us to do so by supplying us with samples. And here it may be noted that we have been informed that Sir Joseph Lister, the pioneer in every antiseptic treatment, is returning to the use of carbolic acid after having largely employed the mercurial and mercurial with zinc methods of antiseptsis. First, we have all the various grades of carbolic acid *per se*, varying from the coarse commercial product for drain disinfection to the absolutely pure drug for internal medicinal use. Then we have the acid in combination with soaps, and these are in great variety. Amongst them we note a carbolic coarse soap for scrubbing floors, &c.; ditto dog soap, 10 per cent. Toilet soaps, from the 20 per cent. transparent soap for disinfection after handling infectious persons or things, to the delicate perfumed toilet and nursery soap, which is suitable not only for the common washes of infants and children, but also for general toilet purposes. Besides these there is a soft soap, which, when mixed with water, makes an excellent application to rose and other plants in order to destroy aphides, lice, etc. It can be used, too, to destroy ticks, fleas, and other insect pests in dogs and cattle. Whilst on the subject of soaps we may first notice this firm's paraffin soap. Dissolved in hot water, it removes the grease and dirt from clothes with great rapidity, and without the toil and trouble of scrubbing. We have proved this experimentally. Taking a black gun-flannel, well greased by many weeks of usage, we boiled it with some of the soap, and were much astonished to find all the removable dirt and grease quite gone, no marks remained except the old rust marks, which nothing less than mineral acid would take out. A stronger proof could not be quoted. We believe that this soap is largely used in hospitals. Finally, we note the carbolic powder for disinfectant purposes. Shaken over the contents of dust-bins and in stables, out-houses, or anywhere where active putrescence, with its consequent ill-odours is taking place, it quickly puts an end to these processes and their evil smells. We have stated nothing about the purity, &c., of the samples submitted to our investigation. Wherever carbolic acid is employed it may be practically stated Calvert's is the preparation used. The firm's reputation is so well known that we entered on our sample testing with a strong prepossession in their favour. Nor were we disappointed. The samples were fully up to the strength and qualities named on their wrappers.

